# Inhibition of cell expansion enhances cortical microtubule stability in the root apex of *Arabidopsis thaliana*

**DOI:** 10.1186/s40709-021-00143-8

**Published:** 2021-06-03

**Authors:** Veronica Giourieva, Emmanuel Panteris

**Affiliations:** 1 Department of Botany, School of Biology, Aristotle University of Thessaloniki, 541 24 Thessaloniki, Greece; 2 Laboratory of Biochemistry, School of Chemistry, Aristotle University of Thessaloniki, 541 24 Thessaloniki, Greece

**Keywords:** *Arabidopsis thaliana*, Cell expansion, Cell wall, Cellulose Synthase A, Colchicine, Cortical microtubules, Oryzalin

## Abstract

**Background:**

Cortical microtubules regulate cell expansion by determining cellulose microfibril orientation in the root apex of *Arabidopsis thaliana*. While the regulation of cell wall properties by cortical microtubules is well studied, the data on the influence of cell wall to cortical microtubule organization and stability remain scarce. Studies on cellulose biosynthesis mutants revealed that cortical microtubules depend on Cellulose Synthase A (CESA) function and/or cell expansion. Furthermore, it has been reported that cortical microtubules in cellulose-deficient mutants are hypersensitive to oryzalin. In this work, the persistence of cortical microtubules against anti-microtubule treatment was thoroughly studied in the roots of several *cesa* mutants, namely *thanatos*, *mre1*, *any1*, *prc1-1* and *rsw1*, and the Cellulose Synthase Interacting 1 protein (*csi1)* mutant *pom2-4*. In addition, various treatments with drugs affecting cell expansion were performed on wild-type roots. Whole mount tubulin immunolabeling was applied in the above roots and observations were performed by confocal microscopy.

**Results:**

Cortical microtubules in all mutants showed statistically significant increased persistence against anti-microtubule drugs, compared to those of the wild-type. Furthermore, to examine if the enhanced stability of cortical microtubules was due to reduced cellulose biosynthesis or to suppression of cell expansion, treatments of wild-type roots with 2,6-dichlorobenzonitrile (DCB) and Congo red were performed. After these treatments, cortical microtubules appeared more resistant to oryzalin, than in the control.

**Conclusions:**

According to these findings, it may be concluded that inhibition of cell expansion, irrespective of the cause, results in increased microtubule stability in *A. thaliana* root. In addition, cell expansion does not only rely on cortical microtubule orientation but also plays a regulatory role in microtubule dynamics, as well. Various hypotheses may explain the increased cortical microtubule stability under decreased cell expansion such as the role of cell wall sensors and the presence of less dynamic cortical microtubules.

**Supplementary Information:**

The online version contains supplementary material available at 10.1186/s40709-021-00143-8.

## Background

Plant cell growth and morphogenesis are directed by cellulose microfibril orientation, which is controlled by the cortical microtubule array (among others see [1–3]). The regulatory role of cortical microtubules on cellulose microfibrils was initially postulated, and remains widely accepted, by the “alignment hypothesis” [4]. According to it, cellulose microfibrils run parallel to cortical microtubules, which guide the movement of Cellulose Synthase A (CESA) complexes (CSCs) [4, 5]. However, cortical microtubules reside just under the plasma membrane, while cellulose microfibrils are synthesized by transmembrane CSCs [6], consisting of CESA subunits. Specifically, the *Arabidopsis thaliana* genome encodes for 10 *CESA* isotypes [7, 8]. *CESA1*, *CESA3* and *CESA6* genes are essential in primary cell wall synthesis [9–11]. Three additional isoforms, the CESA2, CESA5 and CESA9 show sequence and functional redundancy with CESA6 subunit [12, 13], while CESA4, CESA7 and CESA8 are required for cellulose deposition in secondary walls [7, 8]. Mutations in any of the CESA subunits, involved in primary wall synthesis, result in defective cellulose deposition, root radial swelling and reduced cell expansion [9, 10, 14, 15].

Even though the “alignment hypothesis” has been proposed decades ago, a mechanism explaining how cortical microtubules, located inside of the plasma membrane, control the orientation of cellulose microfibrils, outside of the protoplast, remained ambiguous. Several models have been proposed to answer the above-mentioned question [16–18]. The direct observation of CESA complexes sliding on the plasma membrane over the cortical microtubules [5] directly supported the model of Heath [16]. Additionally, it has been recently found that cortical microtubules are connected to CESA complexes via the Cellulose Synthase Interacting 1 (CSI1) protein [19–21] and Companion of Cellulose Synthase protein 1 and 2 (CC1 and CC2), respectively [22].

Apart from the role of cortical microtubules on cellulose microfibril orientation, several authors supported that CESA activity and cellulose synthesis also exert an effect on cortical microtubule orientation, suggesting thus that cortical microtubule-cellulose microfibril relationship is bi-directional. In particular, experiments with cellulose synthesis inhibitors [23] and genetic analysis of CESA mutants (*cesa*) revealed that CESA impairment and cellulose deficiency affect cortical microtubule stability and organization (among others [24–26]). In addition, Le et al. showed that exposure to ethylene inhibited cell elongation in *A. thaliana* root tips and consequently altered cortical microtubule orientation [27]. Thorough experiments by Panteris et al. have revealed that the primary factor for microtubule disorientation in mutants with defective cellulose synthesis and plants affected by cellulose synthesis inhibitors is the suppression of cell expansion, which is a direct consequence of cellulose shortage [28, 29].

Even though plenty of data about cortical microtubule orientation and cell wall mechanics are now available, information about the possible relationship of cellulose synthesis and/or cell expansion with cortical microtubule stability, manifested as sensitivity or resistance to depolymerizing drugs, remains scarce. Paredez et al. have shown that *prc1-20*, an *A. thaliana cesa6* mutant, is more sensitive to the microtubule depolymerizing drug oryzalin, than the wild-type [25]. A similar observation has been also reported for the CSI1 mutant, *csi1-2*, which is defective in cellulose synthesis [30].

In order to further investigate if, apart from *prc1-20* [25], mutations in other *A. thaliana CESA* genes affect the sensitivity of cortical microtubules to oryzalin, *cesa1*, *cesa3* and *cesa6* mutants were examined in the present study. Surprisingly, the cortical microtubules, in the root cells of the mutants reported here, appeared more persistent against oryzalin, compared to the microtubules of the wild-type. Furthermore, to dissect if this increased persistence is due to defective cellulose synthesis or to decreased cell expansion, chemical treatments with DCB (2, 6 dichlorobenzonitrile; inhibitor of cellulose synthesis, [31]) or Congo red (cellulose binding stain; [32, 33]) were also performed. The observations support that the increased persistence of cortical microtubules is associated with inhibition of cell expansion rather than cellulose deficiency.

## Methods

### Plant materials and growth conditions

*Arabidopsis thaliana* (L). Heynh wild-type, ecotype Columbia (Col), the *thanatos* (*than*) semi-dominant *cesa3* mutant and wild-type seedlings expressing the *thanatos* allele of *atcesa3* (*Col-0::AtCesA3*(P578S), from now on referred as *pcesa3* [14]), the *mre1* (*multiple response expansion1* [34]), the *any1* (*anisotropy1* [15]), the temperature-sensitive *cesa1* mutant *rsw1* (*radially swollen1* [9]), the *cesa6* mutant *prc1-1* (*procuste1-1* [10]) and the *csi1* mutant *pom2-4* [20] were used in the experiments. Seeds of the aforementioned plant material were surface-sterilized with sodium chlorate 30 % (v/v) and kept in the dark at 4 ^o^C for 48–72 h. The seeds were germinated on modified Hoagland ’s solution (4 mM KNO_3_, 1 mM Ca(NO_3_)_2_, 2 mM KH_2_PO_4_, 0.3 mM MgSO_4_, 0.09 mM Fe-citrate and 1 mL L^− 1^ micronutrients), supplemented with 2% (w/v) sucrose and 1 % (w/v) phytoagar (Duchefa, Haarlem, the Netherlands). Seedlings were grown in Petri dishes placed vertically in a growth chamber at 21 ± 1 °C, photoperiod of 16 h light and 8 h dark and light intensity 120 µmol m^− 2^ s^− 1^.

Wild-type seeds, *rsw1* and *prc1-1* were obtained from NASC (Nottingham Arabidopsis Stock Center, University of Nottingham, UK) while the rest of the above seeds were kindly provided as follows: *thanatos* and *pcesa3* by Dr. Stamatis Rigas, Dr. Gerasimos Daras and Prof. Polydefkis Hatzopoulos; *mre1* by Prof. Leonard Pysh; *any1* by Prof. Geoffrey Wasteneys; *pom2-4* by Prof. Staffan Persson. Chemicals and reagents were purchased from Applichem (Darmstadt, Germany), Merck (Darmstadt, Germany) and Sigma-Aldrich (Seezle, Germany) unless it is referred differently.

### Chemical treatments

Wild-type seedlings, *than*, *pcesa3* and *mre1* were treated by pouring oryzalin solution over the dishes as well as by transplanting in dishes with solid medium supplemented with oryzalin. The rest of the seedlings were treated only *via* transplanting in dishes with drug-supplemented medium. After germination, 5–7 day-old seedlings were treated for 4 or 6 h with oryzalin, either by pouring an aqueous solution of the drug over the seedlings in the Petri dishes, which were continuously shaken on a rocking platform, or by transplanting the seedlings in dishes with medium supplemented with working oryzalin concentration. The final oryzalin concentration was 200 nM (for longer treatment) or 400 nM (for short treatment; readily diluted from a stock solution of 20 mM in dimethyl-sulfoxide). Seedlings of the wild-type as well as of all the mutants, except *rsw1*, were treated at room temperature. Since *rsw1* expresses its cellulose-deficient phenotype at 30 °C, wild-type and *rsw1* seedlings remained for 1 h at a 30 °C chamber before treatment with oryzalin, which was also performed at 30 °C. In addition, wild-type, *than*, *prc1-1* and *any1* seedlings were transplanted to medium containing 2 mM colchicine (a concentration usually applied on plant cells; Panteris et al. and references therein [35]) for 1.5 h.

Furthermore, wild-type seedlings were treated with combined applications of 400 nM DCB (diluted from stock 10 mM in dimethyl-sulfoxide) and 200 nM oryzalin. Specifically, wild-type seedlings grown in Petri dishes were transplanted to medium containing 400 nM DCB for 22 h and afterwards were transplanted to medium containing 400 nM DCB + 200 nM oryzalin for another 6 h. Wild-type seedlings transplanted to medium with 400 nM DCB for 28 h as well as wild-type seedlings transplanted to medium containing 200 nM oryzalin for 6 h were used as control samples for the combined treatments.

The same procedure was followed for the combined treatment with 10 mg L^− 1^ Congo red (G. Grubler & Co., Berlin, Germany) and 200 nM oryzalin. Wild-type seedlings transplanted to medium containing 10 mg L^− 1^ Congo red for 28 h were used as control samples.

### Immunolocalization

Immunolabelling was performed on seedlings untreated or treated as above, with monoclonal rat anti–*α*-tubulin (YOL1/34, AbD Serotec, Kidlington, UK) and FITC–anti–rat (developed in goat, Invitrogen, Carlsbad, CA) as primary and secondary antibody, respectively, diluted in PBS (Phosphate buffered saline, pH 7.4) at 1:40 for whole-mount immunofluorescence microscopy. The protocol described in Panteris et al. was applied [29]. The samples were examined with a Zeiss LSM780 confocal laser scanning microscope (CLSM), images were acquired with ZEN2011 software (Carl Zeiss, Munich, Germany), following the manufacturer’s instructions. Imaging and processing settings were the same for control and treated samples, both wild-type and mutants.

### Fluorescence intensity measurements

Fluorescence intensity quantifications were performed in maximum projections of serial CLSM sections of root tips, in the transition and fast elongation zone [36], using the ImageJ software [37] according to Pappas et al., using the formula for corrected total cell fluorescence (CTCF) [36, 38]. A total of at least thirty individual cells from three root tips per treatment were used for the fluorescence intensity calculation (10 cells (*n*) were measured in each root tip). All experiments were performed in three technical and biological repeats. Statistical significance was analyzed by non-parametric Student’s t-test, with significance level set at *p* < 0.005.

## Results and discussion

### Effects of oryzalin on cortical microtubules in *cesa3* mutants

To determine whether mutations in CESA3 subunit affect cortical microtubule stability, microtubule depolymerization by oryzalin was assessed. In this study, *A. thaliana* root apex was classified into four distinct zones, the meristematic, transition (also referred as distal elongation zone), fast elongation (also known as elongation zone) and growth terminating zone, according to Verbelen et al. [39]. Epidermal cells of the transition and fast elongation zone were studied for cortical microtubule stability, since cell divisions in them are scarce and microtubules are uniformly distributed. Besides, as one of the first effects of oryzalin is the disruption of the cell division-specific microtubule arrays [40–43], the meristematic zone was also monitored to verify whether seedlings were affected by the drug. The features revealing cortical microtubule stability or susceptibility in the transition and fast elongation zone were their integrity, as exhibited by the degree of fragmentation, and their density in each cell, as revealed by the relevant fluorescence intensity measurement. The orientation and density of cortical microtubules in wild-type seedlings, as well as in the heterozygous *than* and homozygous, *rsw1* and *prc1-1* mutants, have been shown in Panteris et al. [28, 29].

**Fig. 1:**
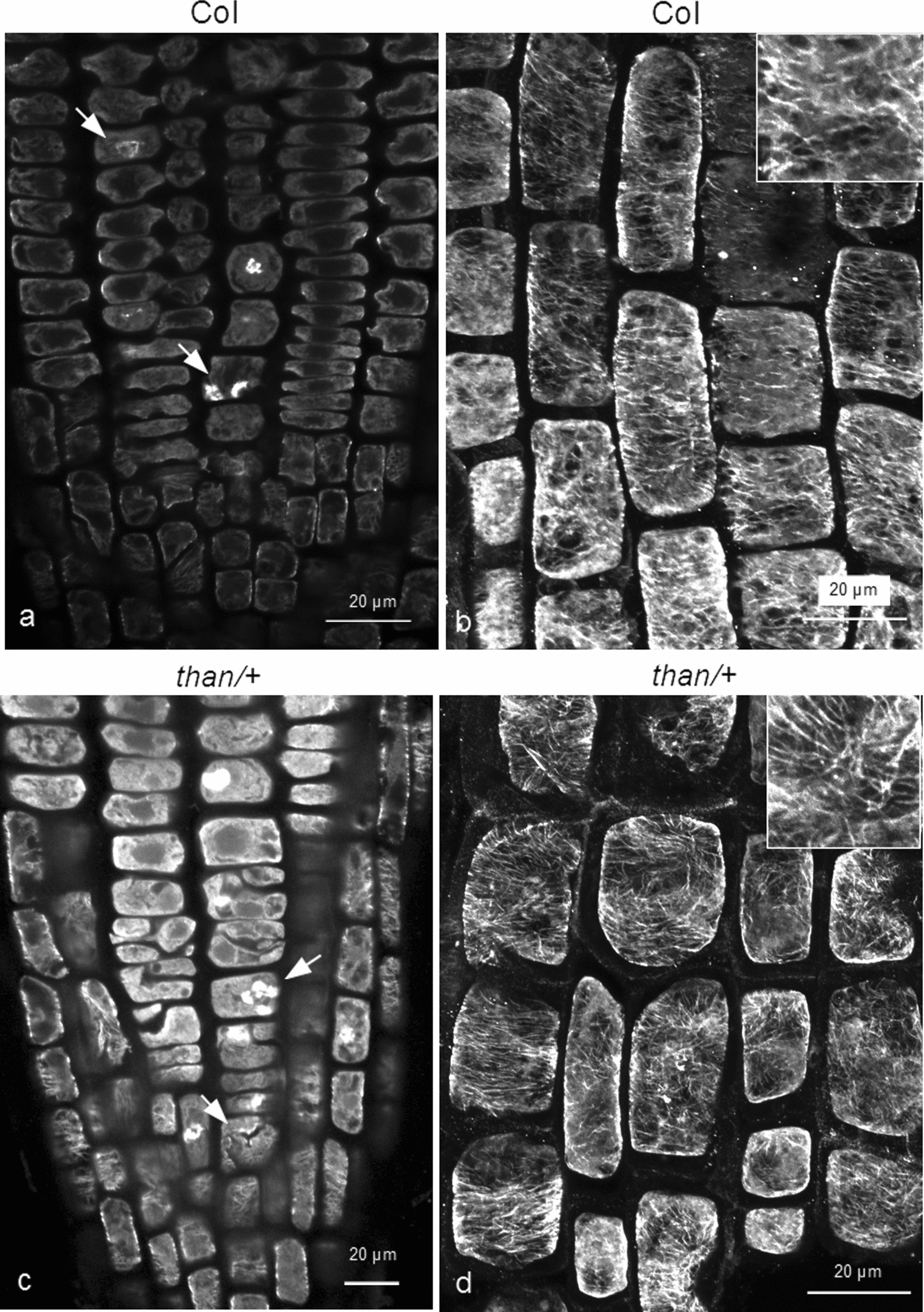
Comparison of microtubule integrity between the wild-type and *than/*+. Effects of oryzalin on 4-5-d-old wild-type (**a**, **b**) and *than/+* (**c**, **d**) roots. Seedlings were treated for 4 h with 400 nM oryzalin. In the meristematic zone, cell divisions are affected (**a**, **c** arrows). Cortical microtubules in transition and fast elongation zone cells of *than/+* appear more integral than those of the wild-type (**d**; cf. **b**). In all the Figures the insets show immunostained microtubules at higher magnification. Scale bars: 20 μm

**Fig. 2:**
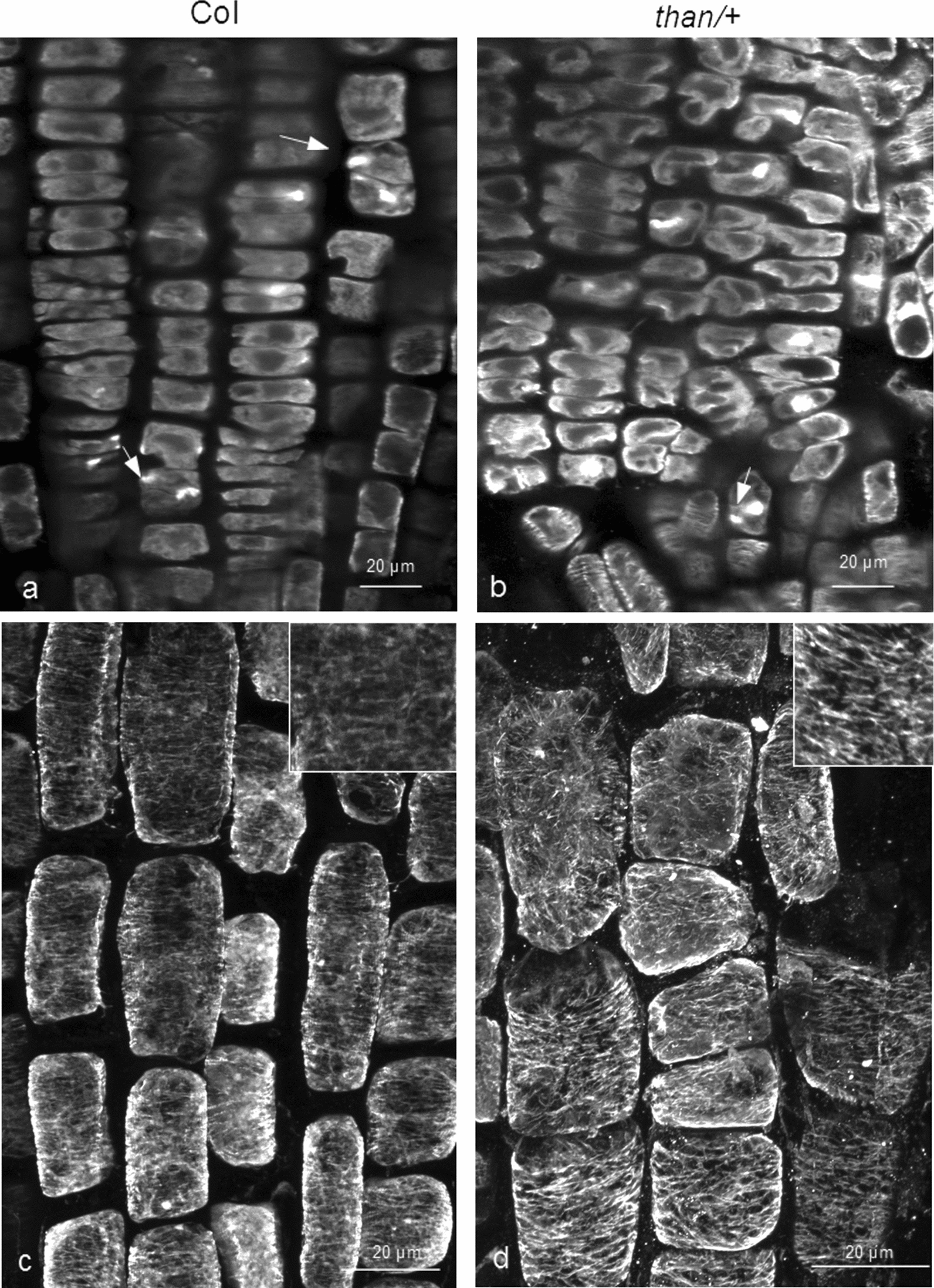
Comparison of microtubule integrity between the wild-type and *than/+****.*** Effects of oryzalin on wild-type (**a**, **c**) and *than/+* (**b**, **d**) roots. Seedlings were transplanted from control medium to substrate with oryzalin 200 nM for 6 h. Cell divisions in the meristematic zone of both wild-type and *than/+* are affected, as incomplete cell walls and abnormal phragmoplasts can be observed (**a**, **b** arrows). Cortical microtubules are longer and more integral in transition and fast elongation zone cells of *than*/+ compared to wild-type roots (**d**; cf. **c**). Scale bars: 20 μm

**Fig. 3:**
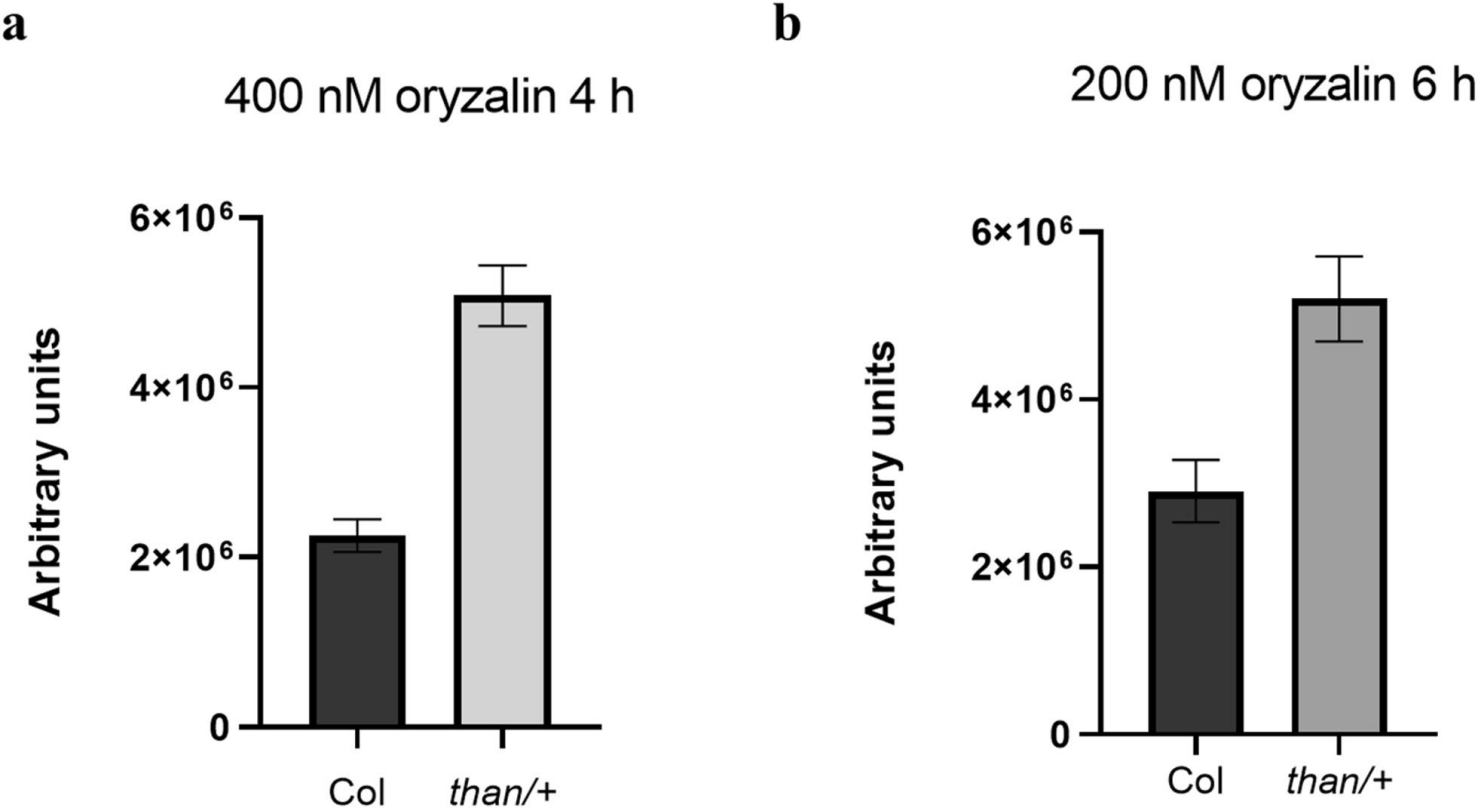
Fluorescence intensity measurements of cortical microtubules in the transition and fast elongation zone of wild-type and* than/+* roots. Maximum intensity projections of serial CLSM sections of transition zone and fast elongation zone were used. Increased fluorescence intensity in *than/+* was observed. (**a**) Effect of 4 h treatment with 400 nM oryzalin. (**b**) Effect of oryzalin on roots transplanted from control medium to substrate with oryzalin 200 nM for 6 h. Error bars indicate standard error. The intensity difference was statistically significant compared to control group (oryzalin-treated Col) (t-test, *p* < 0.001, *n* = 10). In all the figures the *n* corresponds to the number of cells measured in each root tip. At least three root tips were used for fluorescence intensity measurements

Firstly, we investigated cortical microtubule stability in heterozygous *than* (*cesa3*; referred to as *than/+*) seedlings. Cortical microtubules of *than/+* roots, under any procedure of treatment (see Methods), appeared more stable than those of the wild-type (Figs. 1 and 2). In the meristematic zone of both wild-type and *than/+*, abnormal cell divisions, disoriented phragmoplasts and incomplete cell walls could be observed (Figs. 1a and c and 2a, b). In transition and fast elongation zone cells of *than/+* roots, cortical microtubules were more integral and densely arranged, compared to those of the wild-type (Figs. 1b, d and 2c, d). The integrity of cortical microtubules was further verified by the fluorescence intensity measurements (Fig. 3). Taken together, cortical microtubules appear more stable in *than/+* root cells than in those of the wild-type. This indicates that there might be a correlation between cortical microtubule stability and CESA function or cellulose content, as *than/+* is characterized by reduced cellulose content and whole plant dwarfism [14].

**Fig. 4:**
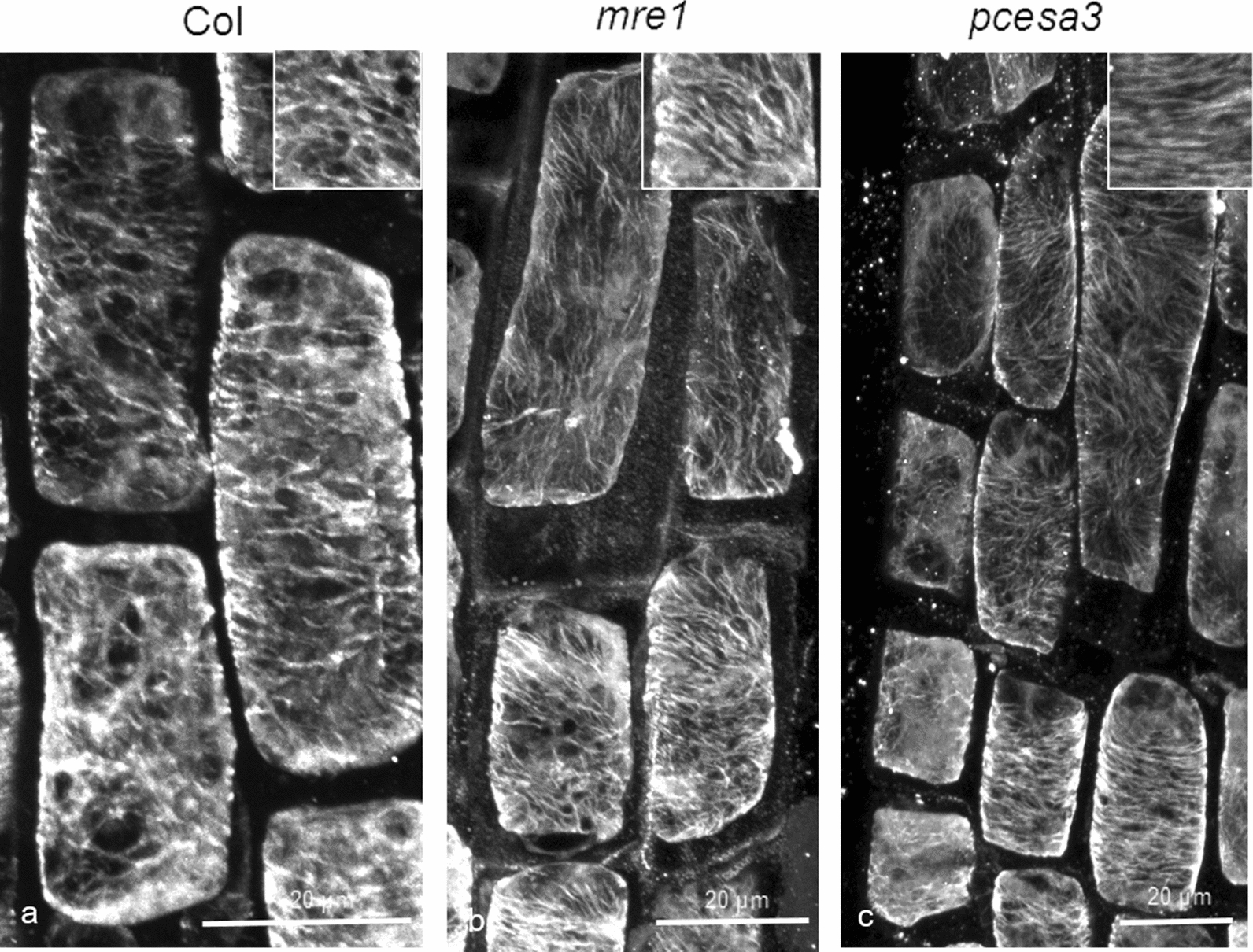
Comparison of microtubule integrity between the wild-type and *mre*1 and *pcesa3*. Effects of 4 h treatment with 400 nM oryzalin on 4-5-d-old wild-type (**a**), *mre1* (**b**) and *pcesa3* (**c**) roots. In both mutants (**b**, **c**) cortical microtubules of transition and fast elongation zone cells appear longer and more integral, compared to those of wild-type roots (**a**). Scale bars: 20 μm

Subsequently, we investigated whether the increased resistance to oryzalin is restricted to the *than/+* mutant only, or it is a general feature of *cesa3* mutants. Therefore, the *mre1* as well as *pcesa3*, a transgenic plant expressing the *than* allele [14], were treated with 400 nM oryzalin for 4 h. Both mutants exhibited cortical microtubules more persistent against oryzalin than the wild-type. Specifically, in transition and fast elongation zone cells of the mutants, cortical microtubules appeared longer and more integral (Fig. 4b, c) than those of wild-type roots (Fig. 4a). In addition, higher fluorescence intensity, as a result of more tubulin polymers, was observed in the mutants, compared to the wild-type (Additional file 1: Fig. S1), which confirms the presence of more and longer microtubules. These observations indicate that cortical microtubule stability is a common feature among *cesa3* mutants.

### **The effect of oryzalin on cortical microtubules in***cesa1* and *cesa6* mutants

**Fig. 5:**
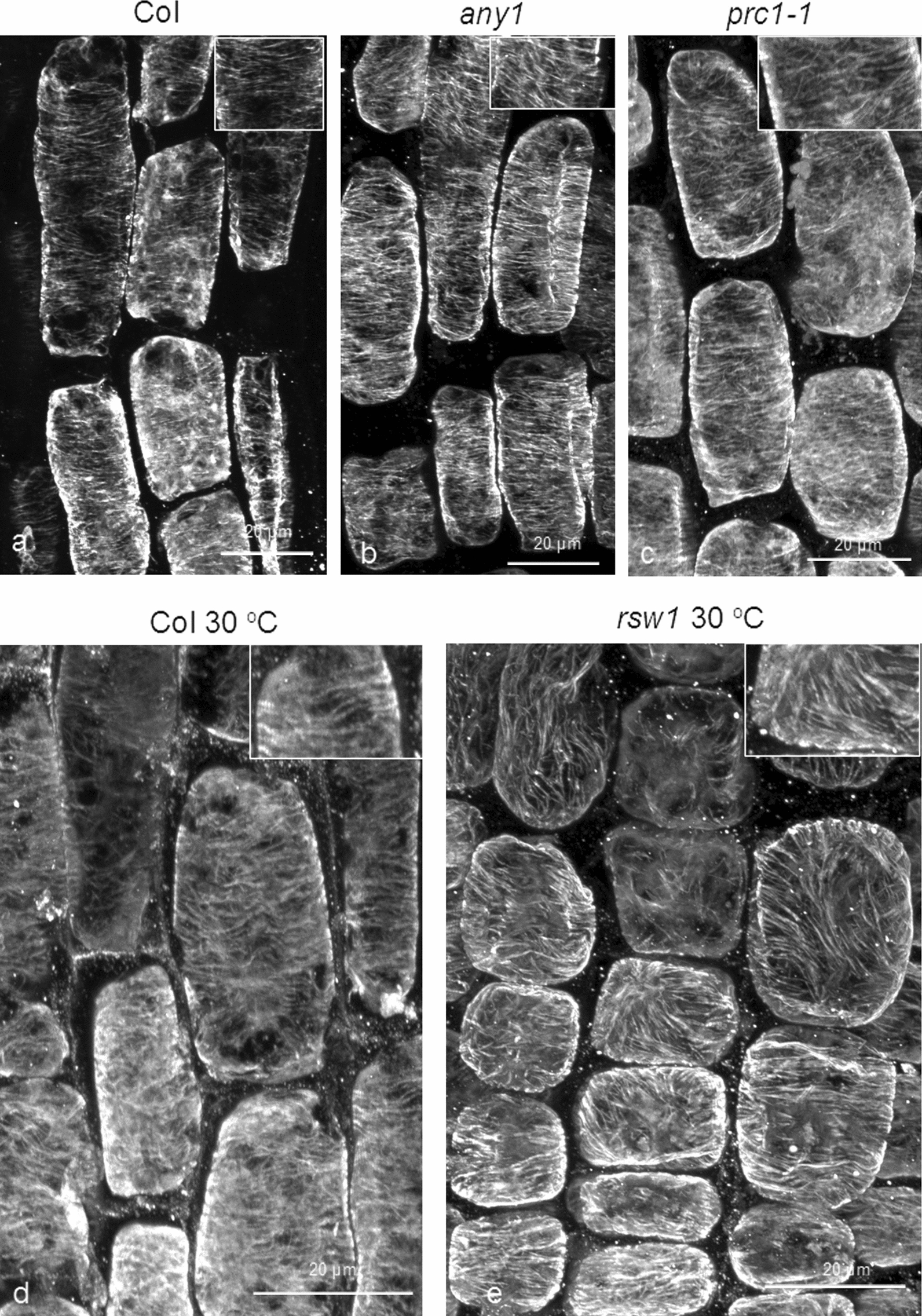
Comparison of microtubule integrity between the wild-type and* cesa1* and* cesa6* mutants. Effects of 6 h 200 nM oryzalin treatment after transplantation of 5-7-d-old wild-type, *any1*, *prc1-1* and *rsw1* seedlings to medium containing the drug. Seedlings in (**d**) and (**e**) were treated at 30 ^o^C, while the others at room temperature. Cortical microtubules in *any1* and *prc1-1* appear less affected, compared to the wild-type (**b** and **c**, compare to **a**). In *any1* and *prc1-1* cortical microtubules are longer and more densely arranged, compared to the wild-type. In *rsw1* cortical microtubules appear more integral than in the wild-type at 30 ^o^C. Scale bars: 20 μm

In order to examine whether cortical microtubule stability is limited to *cesa3* mutants or it might be a general feature of primary cell wall-related *cesa* mutants, *rsw1* and *any1* (*cesa1* mutants), as well as *prc1-1* (*cesa6* mutant) seedlings were treated with 200 nM oryzalin for 6 h. In the transition and fast elongation zones of *any1* and *prc1-1*, more integral microtubules could be observed (Fig. 5b, c), compared to those of wild-type roots. At 30 °C, root cells of *rsw1* showed reoriented but integral cortical microtubules, after treatment with oryzalin, while wild-type roots exhibited fewer and shorter microtubules under the same treatment (Fig. 5e; cf. 5d). The increased persistence of cortical microtubules against oryzalin in the mutants was also confirmed by fluorescence intensity quantification (Additional file 2: Fig. S2). It can, therefore, be concluded that increased cortical microtubule persistence against oryzalin is a common feature of primary cell wall-related *cesa* mutants.

Our results are different from those of Paredez et al., who described the *prc1-20* (*cesa6* mutant) as hypersensitive to oryzalin [25]. This statement was supported by reorientation of cortical microtubules in root cells, as well as by root swelling when exposed to oryzalin. The differences between our findings and the above may be due to different observation approaches and/or the specific criteria for assessing stability. In the above work [25], a GFP-MAP4 marker was used to visualize microtubules, while here *α*-tubulin immunostaining was performed. Microtubule-Associated Protein 4 (MAP4) decorates microtubules and its fusion to GFP allows microtubule visualization [24]. It has been considered that overexpression of GFP-MAP4 fusion proteins may produce an altered developmental phenotype of microtubule dynamics and orientation [24, 44]. In contrast to GFP markers, immunostaining provides more precise and exact microtubule phenotype as it does not interfere with expression and/or function.

In addition, Paredez et al. did not observe any microtubule disassembly but reorientation [25]. In our study, however, cortical microtubules underwent depolymerization without reorientation. In terms of the properties of anti-microtubule drugs, this behavior appears more expected [40] than microtubule reorientation.

### The effect of colchicine on cortical microtubules of *cesa1*, *cesa3* and *cesa6*

**Fig. 6:**
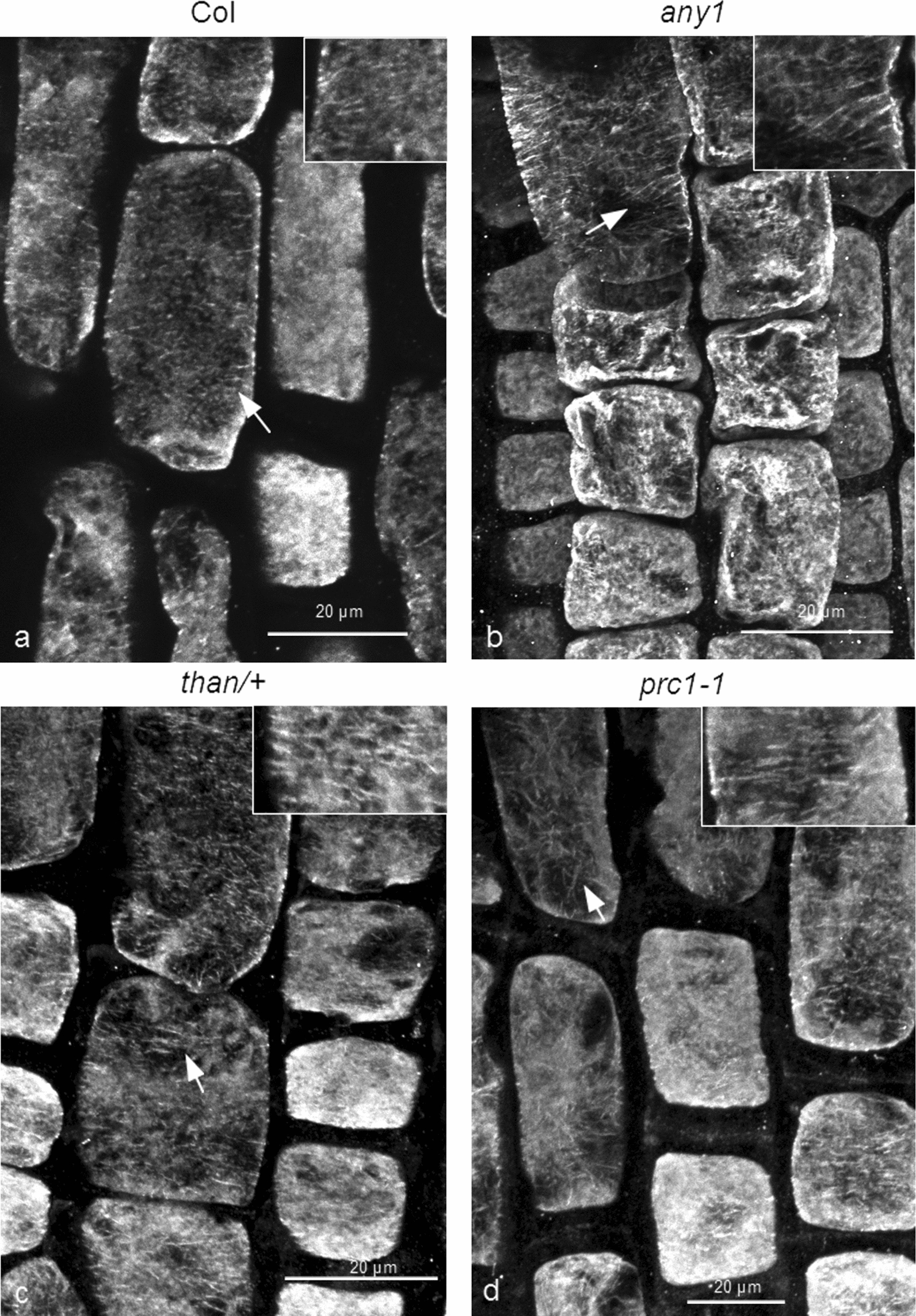
Effects of colchicine on 7-d-old wild-type (**a**), *any1* (**b**), *than*/+ (**c**) and *prc1-1* (**d**). Transition and fast elongation zone cells were examined, after transplantation to medium containing 2 mM colchicine for 1.5 h. Cortical microtubules are extensively disrupted and only remnants can be observed in some cells (arrows). These microtubule remnants are longer and exhibit higher density in *any1*, *than/+* and *prc1-1* cells, compared to those of wild-type roots (**b**–**d**, compare to **a**). Scale bars: 20 μm

In order to further investigate whether the increased cortical microtubule stability of the above *cesa* mutants is limited to oryzalin or it is generally manifested against anti-microtubule drugs, treatments with colchicine, another microtubule-depolymerizing drug were administered. Colchicine is the most “classic” and “notorious” drug that disrupts microtubules (among others [45, 46]). Wild-type, *any1*, *than/+* and *prc1-1* seedlings were treated with 2 mM colchicine for 1.5 h. The concentration of the drug applied was much higher than that of oryzalin, since colchicine was reported to be effective on plants at higher concentrations (among others [45, 47]). All the seedling roots affected by colchicine showed extensive microtubule depolymerization (Fig. 6). The microtubule remnants were scarce and short. However, the fragments of microtubules appeared longer in the mutants than in the wild-type (Fig. 6b–d, cf. 6a). These results were further verified by the fluorescence intensity measurements (Additional file 3: Fig. S3). Increased cortical microtubule stability may thus be considered as a general feature among *cesa* mutants, irrespective of the drug or the CESA subunit mutation.

### Cortical microtubule stability in the *pom2-4* mutant

**Fig. 7:**
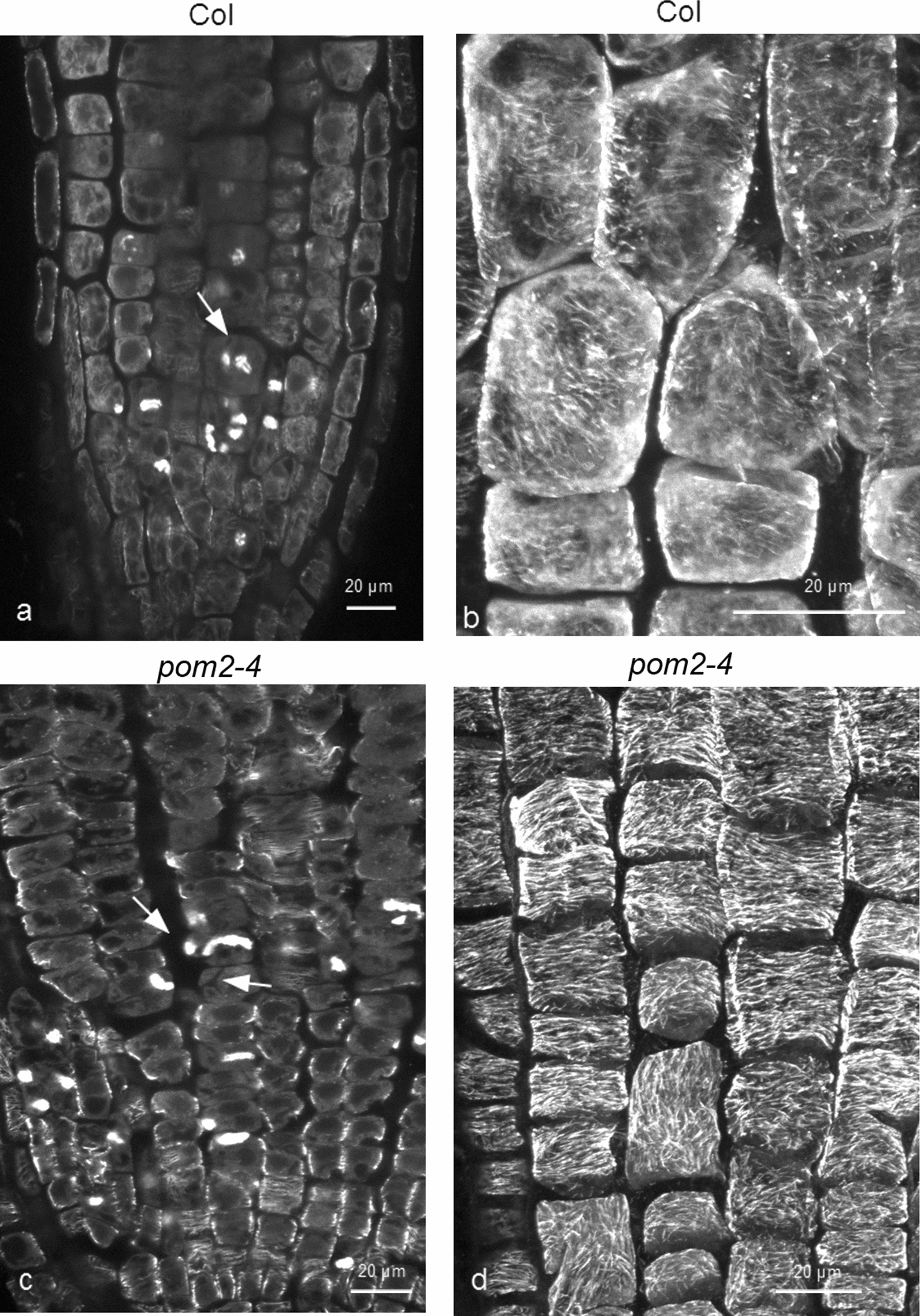
Comparison of microtubule integrity between the wild-type and *pom2-4*. Microtubule integrity in 5-d-old wild-type (**a**, **b**) and *pom2-4* (**c**, **d**) roots, after transplantation to medium containing 200 nM oryzalin for 6 h. Cell divisions in the meristematic root zone are affected by the drug (arrows in **a** and **c**). In transition and fast elongation zone cells, *pom2-4* exhibits longer and more integral cortical microtubules (**d**), in comparison to the wild-type, the microtubules of which appear depolymerized and display intense background fluorescence (**b**). Scale bars: 20 μm

So far, mutants with deficient CESA subunits and decreased cellulose synthesis, except *any1*, which shows reduced cell wall crystallinity and CESA velocity but physiological cellulose content [15], have been studied. In order to discriminate if the increased microtubule stability resulted from malfunction of the CESAs *per se* or from defective cellulose synthesis, the *csi1* mutant *pom2-4* was treated with oryzalin. In this *csi1* mutant, defects in growth and cell expansion occur due to decreased cellulose content, although the CESA subunits are normal [19, 20, 48]. After treatment with 200 nM oryzalin for 6 h, cell divisions appeared affected in the meristematic zone of both wild-type and *pom2-4* roots (Fig. 7a, c). In transition and fast elongation zone of roots treated as above, cortical microtubules of *pom2-4* appeared longer and more integral, in comparison with those of the wild-type (Fig. 7d, cf. 7b). Increased fluorescence intensity was observed in *pom2-4*, as well (Additional file 2: Fig. S2). Contrarily to our results, Mei et al. reported that cortical microtubules in hypocotyl cells of *csi1-2* were more sensitive to oryzalin than those of the wild-type [30]. However, their observations were made by GFP-MAP4 imaging, which may affect microtubule properties and behavior (see above). In addition, root cells may react differently to oryzalin than hypocotyl cells. Taken together, our observations support that increased cortical microtubule stability, which is common in *cesa* and *csi1* mutants, should rather be attributed not to CESA malfunction *per se*, but to decreased cellulose content in the cell wall.

### Inhibition of cellulose synthesis increases cortical microtubule stability in wild-type seedlings

**Fig. 8:**
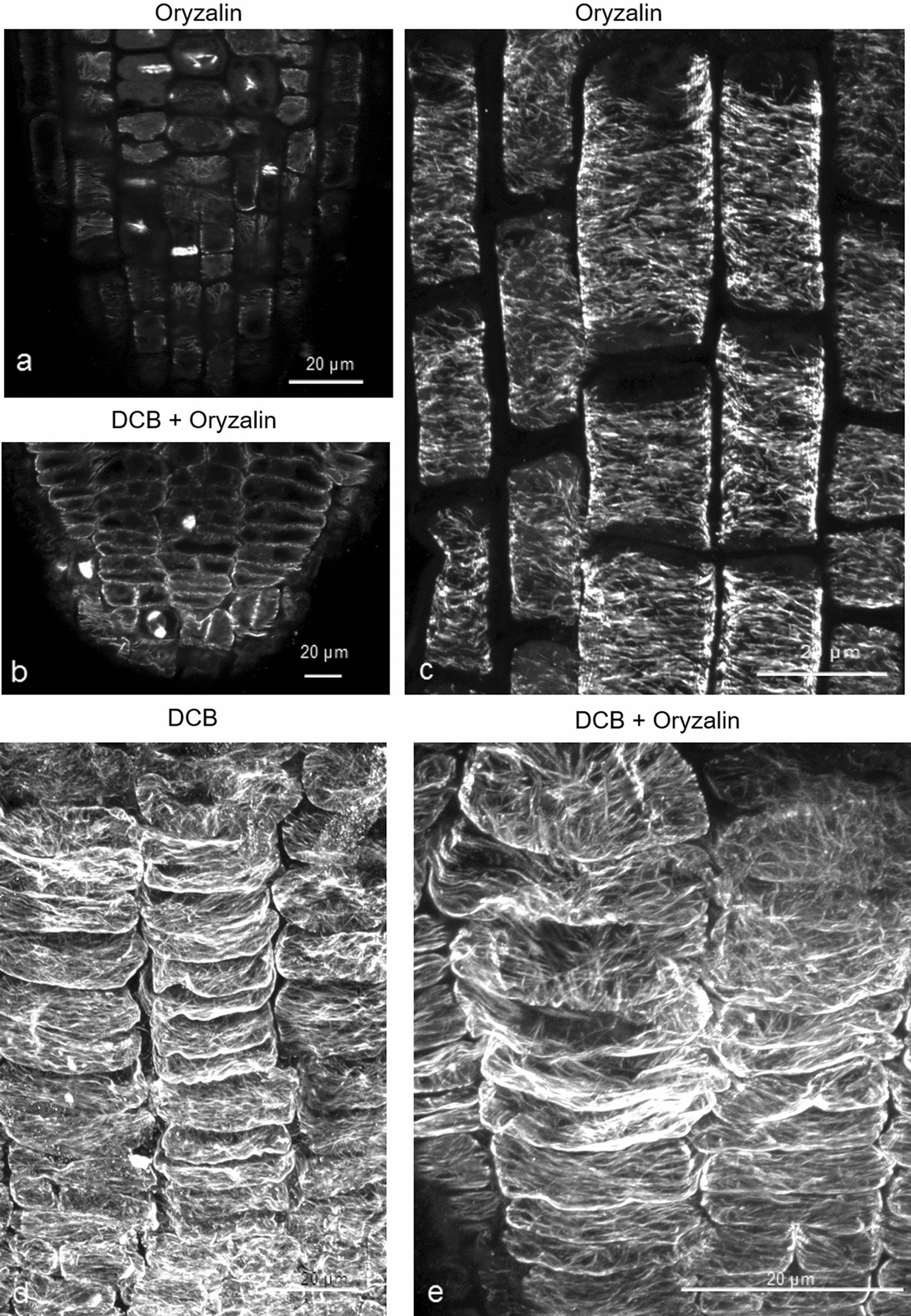
DCB treatment increases microtubule resistance to oryzalin. Effects of combined treatment with DCB and oryzalin on wild-type roots. **a**, **b** Effect of 6 h treatment with 200 nM oryzalin on meristematic zone cells without **a** or after **b** 400 nM DCB treatment. Disordered phragmoplasts and incomplete cell walls can be observed in both cases (**a**, **b**). **c** Oryzalin-affected transition/fast elongation zone cells exhibit disrupted and depolymerized cortical microtubules. **d** Roots of seedlings transplanted for 28 h in medium with 400 nM DCB exhibit short cells with unaffected cortical microtubules. **e** In roots treated for 6 h with 400 nM DCB and 200 nM oryzalin, after been transplanted for 22 h in medium containing 400 nM DCB, cortical microtubules appear unaffected by oryzalin in the transition and fast elongation zone (**e** compare to **d**). Scale bars: 20 μm

**Fig. 9:**
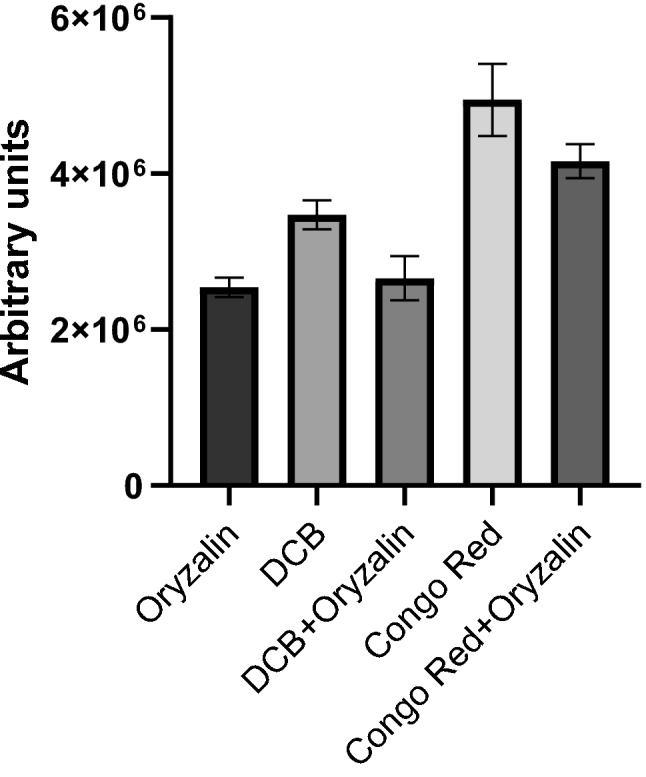
Fluorescence intensity measurements of cortical microtubules of wild-type roots. Prior to 200 nM oryzalin treatment for 6 h, seedlings were transplanted to media containing either 400 nM DCB for 28 h or 10 mg L^− 1^ Congo red for 22 h. Oryzalin-treated seedlings showed the lowest fluorescence intensity. The fluorescence intensity difference was statistically significant for DCB, Congo red and combined treatment of Congo red and oryzalin, compared to the control (*p* < 0.0001, *n* = 10). No statistical significance was observed for intensity fluorescence when oryzalin-treated and combined oryzalin- and DCB-treated roots were compared (*p* = 0.6835, *n* = 10). Error bars indicate standard error

To address the question whether defective cellulose biosynthesis may improve cortical microtubule stability, wild-type roots were examined after combined treatment with DCB and oryzalin. DCB is a synthetic herbicide causing cessation of CESA complex movement, resulting in inhibition of cellulose synthesis and deposition (among others [49, 50]). For this experiment, 4-d-old wild-type seedlings were transferred to a medium containing 400 nM DCB for 22 h and then transferred to medium with 400 nM DCB and 200 nM oryzalin for 6 h. Seedlings treated for 6 h with 200 nM oryzalin alone were used as control (Fig. 8). Root tip bulging and inhibition of seedling growth was observed in DCB and combined DCB and oryzalin treatment (Additional file 4: Fig. S4). Abnormal cell divisions and incomplete cell walls in oryzalin and in the combined treatment prove the effect of oryzalin in the roots (Fig. 8a, b). The length of all root cell types appeared affected after treatment with DCB, and the cells appeared shorter and flattened, compared to untreated root cells (Fig. 8b; cf. 8a, 8d, 8e; cf. 8c). As shown in Fig. 8d, DCB did not affect cortical microtubules. Wild-type roots subjected to combined treatment exhibited cortical microtubules as integral as roots treated with DCB only in the transition and fast elongation zones (Fig. 8e; cf. 8d), not depolymerized as in oryzalin-treated roots (Fig. 8c). Increased fluorescence intensity was observed in DCB and combined DCB and oryzalin treatments compared to the control (Fig. 9).

### Inhibition of cell expansion results in more stable cortical microtubules in the transition and fast elongation zones

**Fig. 10:**
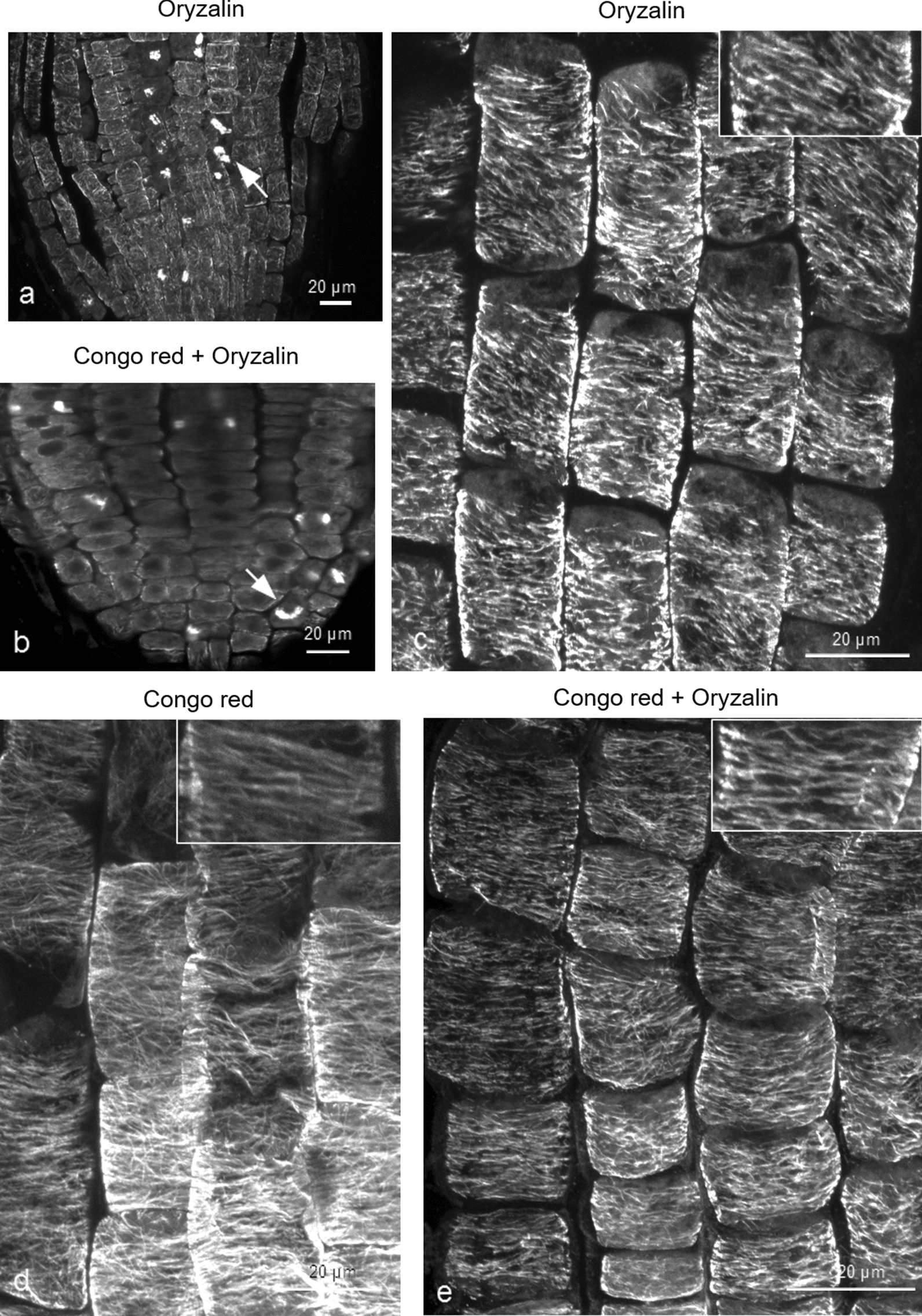
Congo red treatment increases microtubule resistance to oryzalin. Wild-type seedlings treated with Congo red and oryzalin. **a**, **b** Cell divisions (arrows) appear affected in the meristematic zone of roots treated for 6 h with 200 nM oryzalin (**a**) or with 10 mg L^− 1^ Congo red and 200 nM oryzalin for 6 h (**b**). **c** Transition/fast elongation zone cells after 6 h exposure to 200 nM oryzalin exhibit fragmented and partially depolymerized cortical microtubules. **d** Congo red treatment (10 mg L^− 1^ for 22 h) does not affect cortical microtubules. **e** Combined treatment as referred in **b**. Cortical microtubules appear integral, not disrupted as in roots treated with oryzalin alone (**e** compare to **c**). Scale bars: 20 μm

A common feature of cellulose-deficient mutants, as well as of DCB-treated wild-type seedlings, is the inhibition of cell elongation [9, 10, 14, 34, 49]. In addition, although the *any1* mutant of *cesa1* is characterized by normal cellulose content, it exhibits decreased cell elongation due to defective cellulose crystallinity [15]. In order to clarify whether inhibition of cell elongation might be the main cause for increased microtubule stability, the effect of Congo red, a dye that inhibits cell expansion, was investigated (Fig. 10). Congo red, binds to cellulose without affecting its biosynthesis but preventing glucan chain crystallization and, consequently, typical microfibril formation [32, 33, 51]. Wild-type seedlings were treated either with 200 nM oryzalin for 6 h, or they were first transplanted for 22 h to medium supplemented with 10 mg L^− 1^ Congo red and then transplanted to medium containing 10 mg L^− 1^ Congo red and 200 nM oryzalin for 6 h. Root tips of seedlings treated with Congo red or Congo red and oryzalin exhibited intense curling (Additional file 4: Fig. S4). Abnormal cell divisions observed in both oryzalin and combined treatments revealed the depolymerizing effect of oryzalin (Fig. 10a, b). Congo red application resulted in decreased cell elongation in the root apex developmental zones without affecting cortical microtubules (Fig. 10d). After the combined Congo red and oryzalin treatment, cortical microtubules in transition and fast elongation zone cells appeared integral and almost unaffected by oryzalin (Fig. 10e; cf. 10d), in contrast to those of roots treated with oryzalin alone, in which cortical microtubules appeared fragmented and partially depolymerized (Fig. 10c). The increased integrity in Congo red and combined Congo red and oryzalin treatments, compared to the control, was verified by fluorescence intensity measurements (Fig. 9) and was found statistically significant (*p* < 0.0001). According to these findings, it may be concluded that inhibition of cell expansion, irrespective of the cause, results in increased microtubule stability.

Fisher and Cyr were the first to describe a bidirectional relationship between cortical microtubules and the cell wall [23]. According to them, the mechanical properties of cell wall, rather than cellulose biosynthesis, affect cortical microtubule stability and organization. When *Nicotiana tabacum* BY-2 protoplasts were treated with isoxaben (a cellulose biosynthesis inhibitor), microtubules were randomly organized. Cultivation in the absence of isoxaben resulted in highly organized cortical microtubules [23]. Since then, it was supported by several authors that cellulose biosynthesis provides spatial cues for cortical microtubule organization. Chu et al. and Paredez et al. reported alterations in cortical microtubule organization and stability in *cesa* mutants, concluding that cellulose synthesis may play a regulatory role in microtubule organization [24, 25]. On the other hand, Le et al. supported that inhibition of cell elongation in ethylene-treated roots results in microtubule reorientation [27]. In agreement with the latter work, Panteris et al., have demonstrated that chemical, mechanical or genetic inhibition of cell expansion affects cortical microtubule orientation in *A. thaliana* root apex, supporting the bidirectional relationship of cell wall and cortical microtubules [28, 29].

The results of this study support that cell expansion is associated not only with cortical microtubule orientation [28, 29] but also with microtubule stability. The common feature among *cesa* mutants (*any1*, *rsw1*, *than/+*, *mre1*, *prc1-1*), *csi1* mutant (*pom2-4*), DCB and Congo red treatments is that, in all cases, cell expansion is negatively affected. More specifically, decreased cellulose biosynthesis, either caused by CESA malfunction (*any1*, *rsw1*, *than/+*, *mre1*, *prc1-1* and DCB), or by CSI1 (*pom2-4*) mutation, results in inhibition of cell elongation [9, 10, 14, 34, 48–50]. On the other hand, Congo red prevents cellulose crystallization [32, 33], while *any1* shows altered cell wall crystallinity [15]. In conclusion, apart from inducing cortical microtubule reorientation [28, 29], inhibition of cell expansion enhances cortical microtubule stability.

Various hypotheses may explain the increased cortical microtubule stability under decreased cell expansion. It has been reported that cell wall sensors allow cortical microtubules to detect and respond to stress signals [52, 53]. Such sensors include the THESEUS kinase, which is responsible for detecting cellulose deficiency, irrespective the cause, and acting as a cell wall integrity sensor [54]. In addition, another kinase, FERONIA, was reported to be important for cell wall integrity perception, in relation to cell and whole plant growth [55]. According to another hypothesis, it may be possible that, due to decreased cell expansion, cortical microtubules become more stationary. New cellulose microfibrils are deposited during cell elongation, between the existing ones [56]. Concurrently, newly-formed microtubules are recruited to maintain the density of cortical microtubule array. When cell expansion is decreased, the above procedure pertains at slow rate, possibly exerting a pausing effect on cortical microtubules, which remain stable, also interconnected by MAP bridges [57]. As a result, tubulin dimers are not released, while new ones are not added: consequently, tubulin-binding anti-microtubule drugs, such as oryzalin and colchicine exert a less pronounced effect on microtubules.

## Conclusions

This study provides evidence that inhibition of cell expansion, irrespective of the cause, enhanced the stability of cortical microtubules. It is therefore supported that a bidirectional relationship between cell expansion and microtubule stability exists. Further work is required to elucidate the mechanisms underlying this bidirectional relationship.

## Supplementary information

**Additional file 1: Figure S1.** Fluorescence intensity measurements of cortical microtubules in wild type and *cesa3* mutant roots: *mre1* and *pcesa3* roots were treated with 400 nM oryzalin for 4 h. Maximum intensity projections of serial CLSM sections were used. Increased intensity was observed in both mutants, compared to the wild type. Error bars indicated standard error. Fluorescence intensity was measured in totally 30 individual cells of 3 wild type roots and 20 individual cells from 2 roots of *mre1* and *pcesa3* mutants.

**Additional file 2: Figure S2.** Fluorescence intensity measurements of cortical microtubules in wild type, *any1*, *prc1-1*, *pom2-4* and *rsw1* roots. Seedlings were transplanted from control medium to substrate containing 200 nM oryzalin for 6 h. Prior to treatment with oryzalin, some seedlings of the wild type and *rsw1* mutant were incubated at 30 °C for 1 h. Increased fluorescence intensity was observed in all mutants, compared to the respective control. The fluorescence intensity difference for *any1* and *prc1*-*1* was statistically significant compared to control (*p* < 0.0001, *n* = 10) as well for *rsw1* compared to control (*p* < 0.001, *n* = 10). No statistical significance was observed for *pom2-4* compared to control (*p* = 0.13, *n* = 10). The n value corresponds to number of cells measured in each root tip. At least 3 root tips were used for each measurement. Error bars indicate standards errors.

**Additional file 3: Figure S3.** Fluorescence intensity measurements of cortical microtubules in seedling roots treated with colchicine. The wild type and the mutants *any1*, *than/+* and *prc1-1* were transplanted from control medium to substrate supplemented with 2 mM colchicine for 1.5 h. Increased fluorescence intensity was observed in the mutants, compared to the wild type. The *than/+* mutants exhibited the highest fluorescence intensity which was statistically significant compared to treated wild type (*p* < 0.05, *n* = 10). The fluorescence intensity of *any1* and *prc1-1* were not statistically significant compared to control (*p* = 0.12, *n* = 10 for both mutants). The *n* value corresponds to number of cells measured in each root tip. At least 3 root tips were used for each measurement. Error bars represent standard errors.

**Additional file 4: Figure S4.** Effects of chemical treatments (DCB or Congo red and oryzalin) on root growth. 4 to 5-day old wild type seedlings were grown vertically on Petridishes and subsequently transplanted to media supplemented with 400 nM DCB (22 h treatment) or 10 mg L^−1^ Congo red (22 h treatment) and then transferred to media containing additionally 200 nM oryzalin for 6 h. Root tip swelling and curling were observed in DCB and Congo red treated seedlings, respectively. Chemicals were applied as shown in Methods. Scale bar: 1 mm.

## Data Availability

All data generated during this study are included in this published article manuscript and its Additional files.

## Abbreviations

CESA: Cellulose Synthase A
CSC: Cellulose Synthase Complex
CSI1: Cellulose Synthase Interacting 1 protein
CC1: Companion of Cellulose Synthase protein 1
CC2: Companion of Cellulose Synthase protein 2
*prc1-20*: *Procuste1-20*
*than*: *Thanatos*
*than/+*: Heterozygous *than*
DCB: 2, 6 dichlorobenzonitrile.
*rsw1*: *Radially swollen1*
*prc1-1*: *Procuste1-1*
*mre1*: *Multiple response expansion1*
*pcesa3*: Transgenic plant expressing the *than* allele
*any1*: *Anisotropy1*
GFP: Green fluorescent protein
MAP: Microtubule-associated protein
CLSM: Confocal laser scanning microscope
CTCF: Corrected total cell fluorescence
