# Supplementary figures and images for: Inhibition of cell expansion enhances cortical microtubule stability in the root apex of Arabidopsis thaliana
Source: J Biol Res (Thessalon). 2021 Jun 3;28:13. doi: 10.1186/s40709-021-00143-8 (PMC8173746; doi:10.1186/s40709-021-00143-8)

## Slide 1
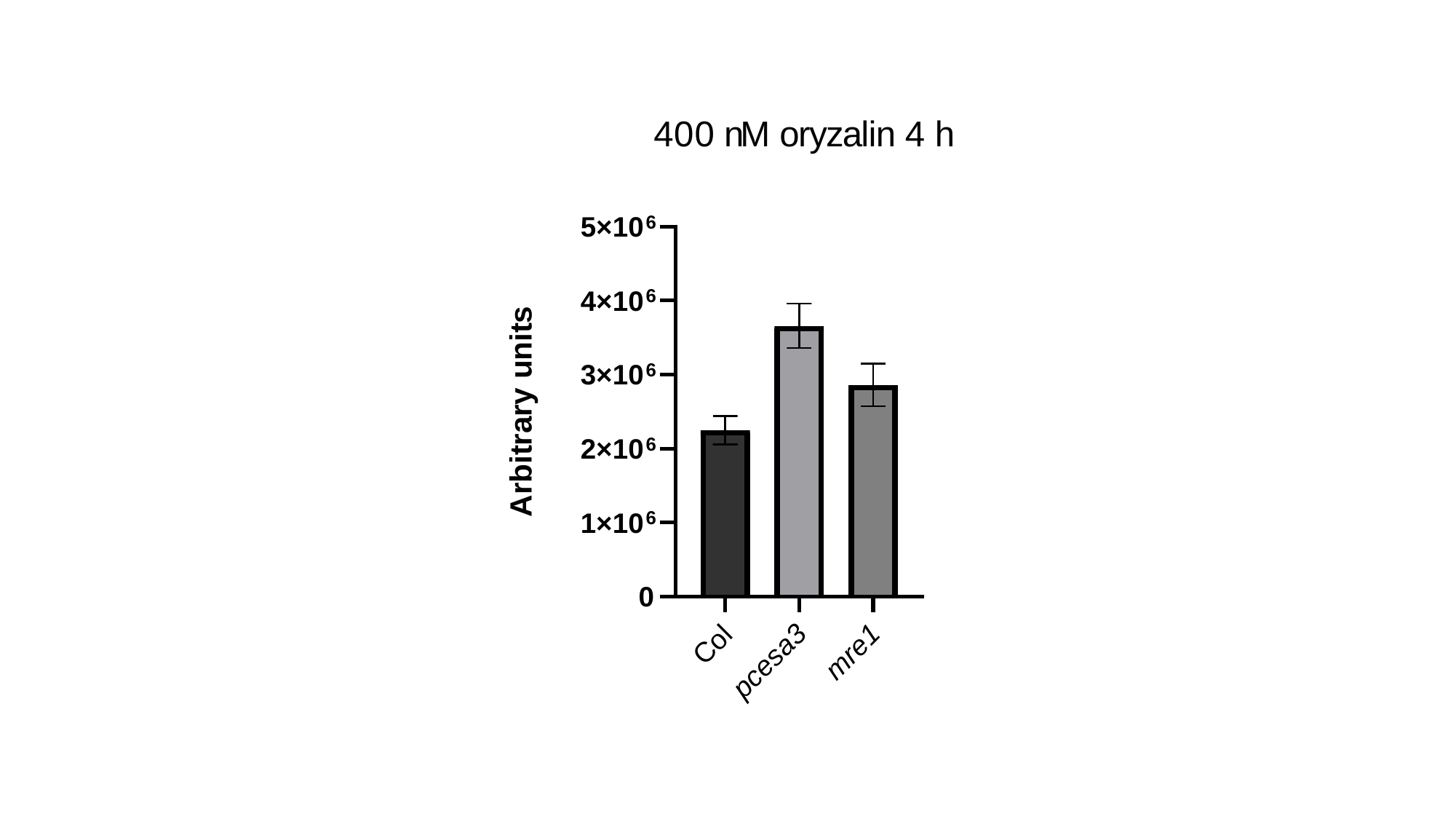

Supplement: Supplementary file 1 — Additional file 1: Figure S1. Fluorescence intensity measurements of cortical microtubules in wild type and cesa3 mutant roots: mre1 and pcesa3 roots were treated with 400 nM oryzalin for 4 h. Maximum intensity projections of serial CLSM sections were used. Increased intensity was observed in both mutants, compared to the wild type. Error bars indicated standard error. Fluorescence intensity was measured in totally 30 individual cells of 3 wild type roots and 20 individual cells from 2 roots of mre1 and pcesa3 mutants. [file 40709_2021_143_MOESM1_ESM.pptx]

## Slide 1
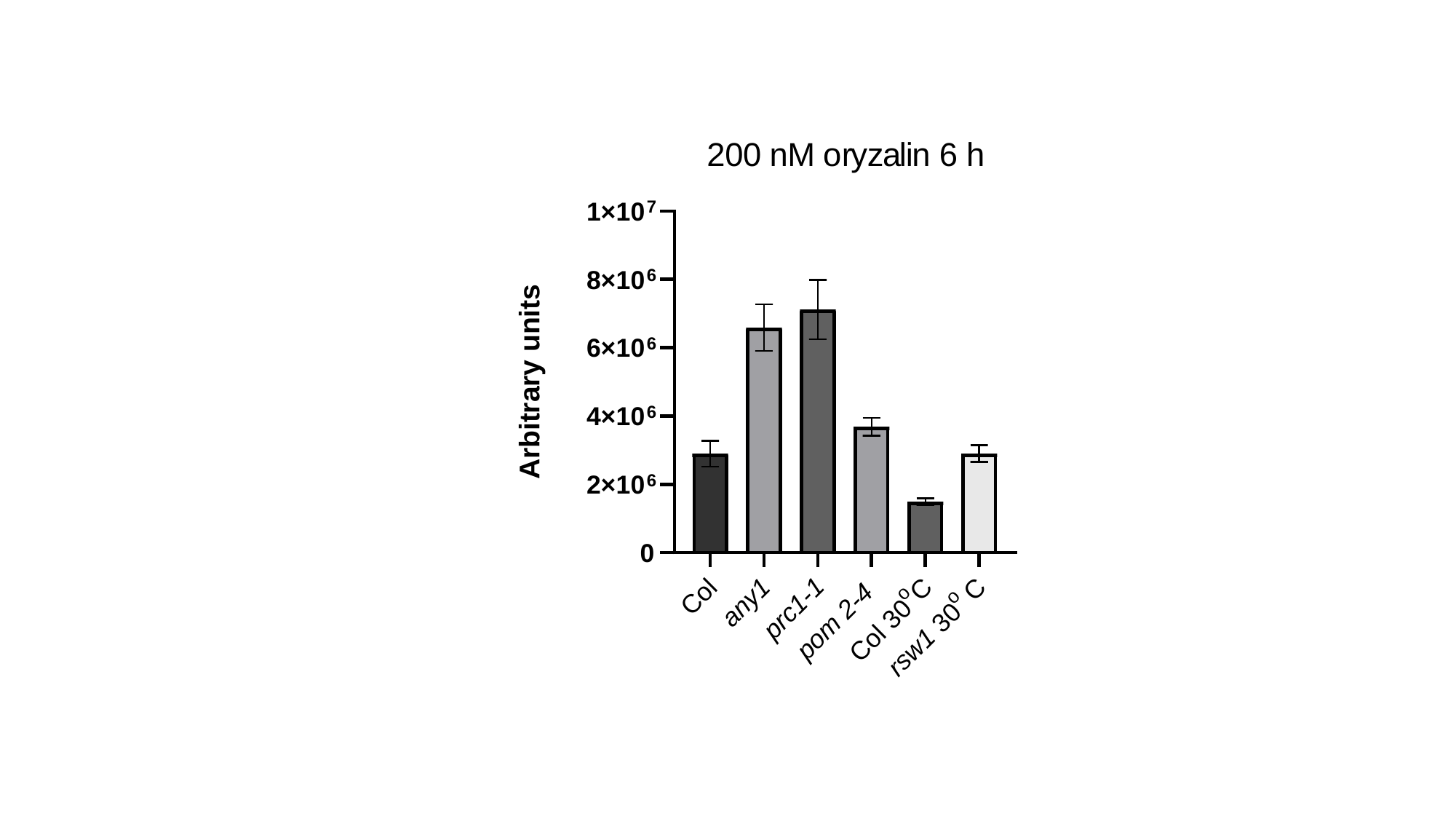

Supplement: Supplementary file 2 — Additional file 2: Figure S2. Fluorescence intensity measurements of cortical microtubules in wild type, any1, prc1-1, pom2-4 and rsw1 roots. Seedlings were transplanted from control medium to substrate containing 200 nM oryzalin for 6 h. Prior to treatment with oryzalin, some seedlings of the wild type and rsw1 mutant were incubated at 30 °C for 1 h. Increased fluorescence intensity was observed in all mutants, compared to the respective control. The fluorescence intensity difference for any1 and prc1-1 was statistically significant compared to control (p < 0.0001, n = 10) as well for rsw1 compared to control (p < 0.001, n = 10). No statistical significance was observed for pom2-4 compared to control (p = 0.13, n = 10). The n value corresponds to number of cells measured in each root tip. At least 3 root tips were used for each measurement. Error bars indicate standards errors. [file 40709_2021_143_MOESM2_ESM.pptx]

## Slide 1
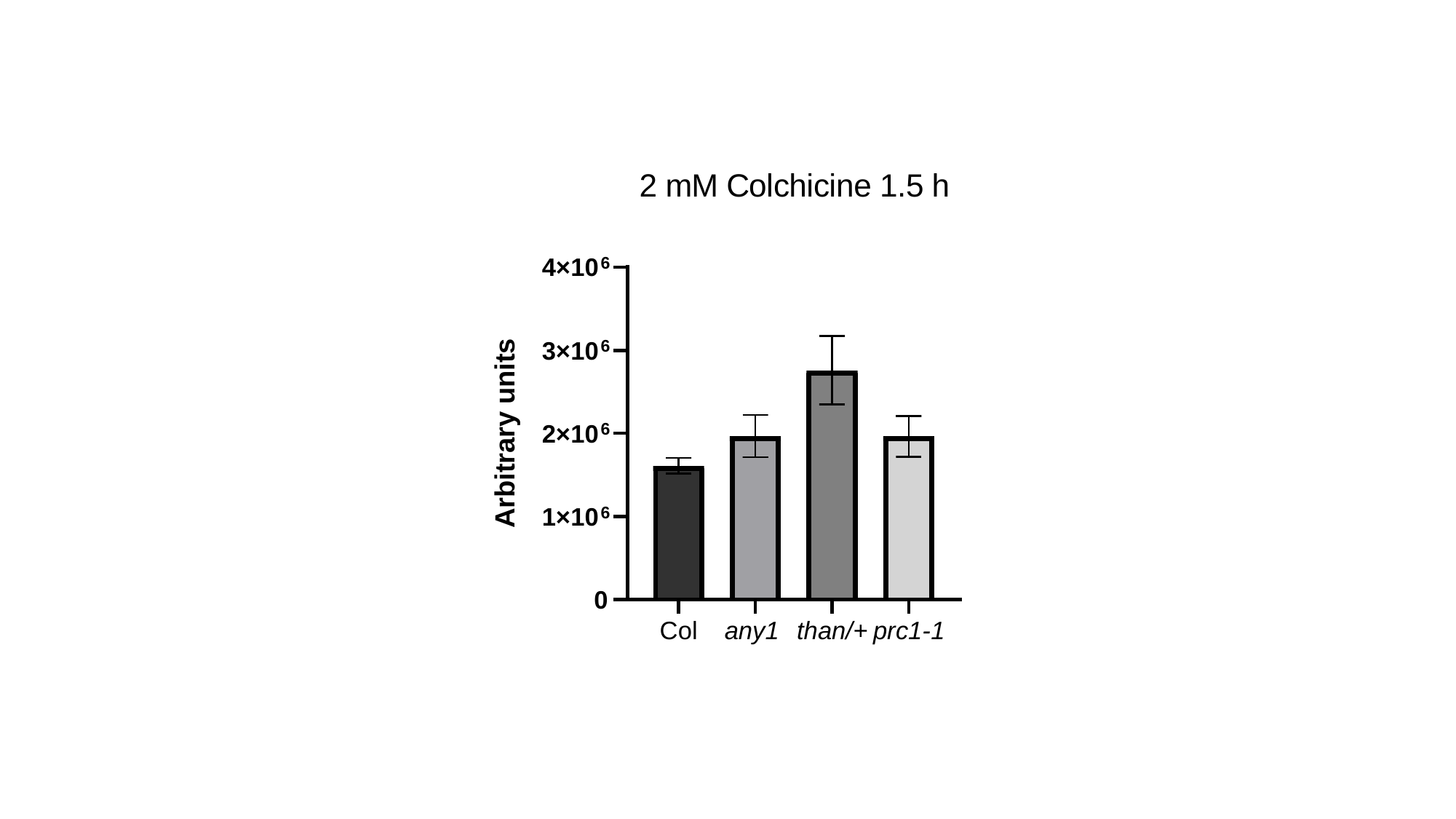

Supplement: Supplementary file 3 — Additional file 3: Figure S3. Fluorescence intensity measurements of cortical microtubules in seedling roots treated with colchicine. The wild type and the mutants any1, than/+ and prc1-1 were transplanted from control medium to substrate supplemented with 2 mM colchicine for 1.5 h. Increased fluorescence intensity was observed in the mutants, compared to the wild type. The than/+ mutants exhibited the highest fluorescence intensity which was statistically significant compared to treated wild type (p < 0.05, n = 10). The fluorescence intensity of any1 and prc1-1 were not statistically significant compared to control (p = 0.12, n = 10 for both mutants). The n value corresponds to number of cells measured in each root tip. At least 3 root tips were used for each measurement. Error bars represent standard errors. [file 40709_2021_143_MOESM3_ESM.pptx]

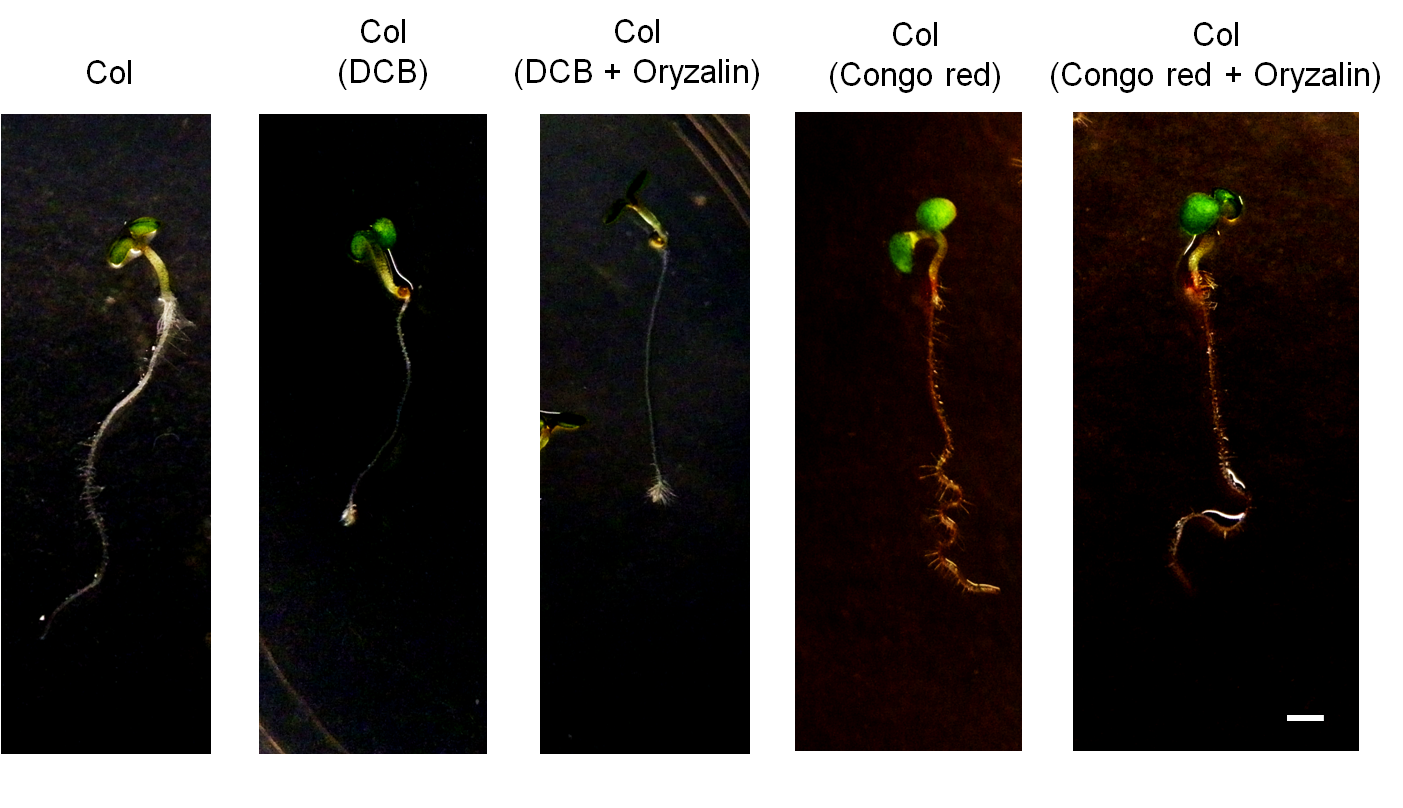

Supplement: Supplementary file 4 — Additional file 4: Figure S4. Effects of chemical treatments (DCB or Congo red and oryzalin) on root growth. 4 to 5-day old wild type seedlings were grown vertically on Petridishes and subsequently transplanted to media supplemented with 400 nM DCB (22 h treatment) or 10 mg L−1 Congo red (22 h treatment) and then transferred to media containing additionally 200 nM oryzalin for 6 h. Root tip swelling and curling were observed in DCB and Congo red treated seedlings, respectively. Chemicals were applied as shown in Methods. Scale bar: 1 mm. [file 40709_2021_143_MOESM4_ESM.tif]
